# A thematic analysis of the perceptions of reversible inhibition of sperm under guidance as a potential family planning method in the United Kingdom

**DOI:** 10.1177/03915603241261813

**Published:** 2024-07-26

**Authors:** Cristina M Gheorghe, Olivia Slack, Amanda D Wilson

**Affiliations:** Faculty of Health and Life Sciences, School of Applied Social Sciences, De Montfort University, Leicester, UK

**Keywords:** RISUG, family planning, new male contraception, United Kingdom, social acceptability, sex differences

## Abstract

**Background:**

This exploratory study aimed to look into public perceptions of Reversible Inhibition of Sperm Under Guidance (RISUG) as a family planning method in the United Kingdom (UK). It also aimed to discover if there were any sex differences in perceptions between males and females.

**Design:**

Twelve semi-structured interviews were conducted, six with males and six with females, all residents of the UK.

**Methods:**

The audio data from the interviews then was transcribed for analysis. An inductive and a semantic thematic analysis was conducted on the data set.

**Results:**

Three main themes were constructed, including: (i) RISUG Hesitancy, (ii) Females perceived benefits of RISUG and (iii) Males perceived concerns regarding RISUG. Hesitancy was related to vaccination hesitancy, females wanted males to have more reproductive autonomy and males placed their concerns through the lens of ‘other’ males that their may be unintended side effects. Together these three themes represent both perceived risk and overall benefits of the method. However, while randomized control trails have been completed to standard for RISUG, males perceived concerns, suggesting a disconnect between the public’s perceptions and professionals understanding of trails.

**Conclusion:**

RISUG was perceived as a viable option for family planning in the future, however trust of the new contraceptive method will need to be fostered among the public in order to effectively transfer knowledge on the potential side effects and the standard of pre-market testing for these. Effective public health messages can result in better education of people concerning the new contraceptive method, including the risks and benefits. By using perceptions to inform health messages around RISUG, researchers and practitioners can learn from potential users how to best address misinformation or concerns, while at the same time building an evidence base for when new male methods reach the contraceptive market.

## Introduction

Reversible Inhibition of Sperm Under Guidance (RISUG) is a contraceptive developed for males.^1^ It is an occlusive co-polymer, formulated of Styrene and Maleic Anhydride (SMA), dissolved in Dimethyl Sulphoxide (DMSO), injectable into both lumens of the vas deferens creating a gel like coating of the inner walls. The gel forms solid precipitates resulting in a positively charged acidic environment that dissolves membranes of mature sperm cells as they pass through the sterilizing environment. The charged and acidic environment denatures the enzymes required to fertilize a mature egg resulting in ejaculation free of fertile sperm, despite sperm being present in the ejaculate.^2^ RISUG is distributed in prefilled micro-syringes estimated to have a 2 year shelf life. In terms of procedure, administration is similar to a non-scalpel vasectomy where after anaesthesia numbs the sites the vas is isolated using a non-penetrating clamp to expose the vas deferens. Once the inguinal region of the vas is expose, both seminal tubes are injected, towards the distal region. Post injection, participants in clinical trials remained under observation for 3 hours and were advised to abstain for 1 week from sexual activity.^3^ There are currently no reports of how to check RISUG has been administered correctly. Benefits of RISUG are that it is a relatively easy, quick and minimally invasive procedure that is also affordable and cost effective.^4^

Phase III clinical trials involved 139 married male participants who had at least two children and received an injection of 120 μl of RISUG.^4^ A 6-month follow-up revealed all participants, and their wives, had normal health and experienced no severe or negative side effects as a result of the polymer’s composition. It is important to note though that a full list of potential severe and negative side effects has yet to be reported. The majority of individuals self-reported experiencing initial scrotal swelling and low grade mild pain, but this was temporary and dissipated within 1 month. However, the researchers do not explain how scrotal pain was treated, leaving little advice for other urologists. In total, 133 of the males experienced either extremely low sperm count or no sperm within 1 month after the date of the injection, with 82.7% of participants achieving azoospermia within 2 months and the remaining participants achieving this within 3–6 months. Six participants did not achieve this due to administration error. Overall, there was a failure rate of 4.3%, which is still lower than typical condom use. Failure rate was reported by the researchers to be a result of manufacturing, with syringes leaking making the required dosage unachievable for administration or as a result of counter punctures of the vas. But it is unclear how many participants could not receive the injection and how many had counter punctures, including how to check for counter puncture. No unintended pregnancies occurred, suggesting RISUG is an effective and safe method of family planning. This study did not attempt to reverse the effects of RISUG, and therefore cannot provide supporting evidence for reversibility. Despite completing trials little is known on the method outside of India where it was developed.

Little evidence exists on perceptions people have towards RISUG as a feasible family planning method in other countries. A current study carried out in the United States (US)^5^ assessed the perceptions and intentions of 460 University students regarding a non-hormonal intra-vasal injectable gel (NH-IVIG) contraceptive, similar to RISUG. Results indicated 28.6% of males and 51.4% of females would be open and likely to use NH0IVIG methods. Positive aspects that appealed to the University students were: ability to depend less on their female partner (for male participants) and an increased partner responsibility (for female participants). Most participants agreed that contraception should be a shared responsibility.

### Aims, objective and research question

RISUG is an under-researched topic, with phase III clinical trials recently ending,^6^ with people unaware of the method. Research that does exist regarding the perceptions investigates the perceptions of a niche group of students, from a single university in the US.^5^ To the authors knowledge this is the first empirical qualitative study on RISUG perceptions. The primary aim of this study was to explore the perceptions of RISUG within the UK. A secondary aim was to begin to explore if there were sex differences in perceptions between males and females. The first objective was to conduct interviews with residents in the UK about their perceptions of RISUG. The second objective sought to stratify the interview sample by recruiting an even number of males and females to allow for a comparison of sex differences to occur. The research question was therefore broad and asked, *What are the perceptions of RISUG as a family planning method within the UK?*

## Methods

### Participants

An opportunistic sampling method, followed by a snowballing sample, was adopted to recruit participants. The total sample consisted of 12 individuals, this sample consisted of 6 male and 6 female subsamples, to allow in depth and rich data. All individuals identified as cisgender, heterosexual residents of the UK, who either were or had been sexually active. The ages ranged from 21 to 31 years of age (for further participant information, see Table 1).

**Table 1.** Participant information table.

Participant pseudonym	Sex	Age
Connor	Male	31
Joe	Male	23
George	Male	22
Ryan	Male	31
Toby	Male	24
Luke	Male	22
Amy	Female	24
Jenna	Female	27
Katie	Female	22
Hannah	Female	21
Emily	Female	26
Sophie	Female	27

### Procedure and ethics

Materials used to recruit participants included: a poster which was distributed among social media platforms Facebook, LinkedIn and Instagram. A linked participant information sheet explained the study; those interested in taking part were provided a consent form requiring signature and a RISUG Education Sheet (see Supplemental Materials) to establish a baseline understanding prior to the interview commencing. Semi-structured interviews were conducted and lasted on average 30 minutes. Participants were made aware that no question was mandatory and that they had a right to withdraw throughout the interview. Once the study concluded, participants were provided with a debrief including support resources. Interviews were audio recorded and transcribed in full.

Ethical approval was obtained from De Montfort University. To guarantee participants were recruited in line with British Psychological Society’s Code of Conduct (2018).^7^ Each participant was assigned a pseudonym and anything identifying mentioned throughout the interviews were further anonymized.

### Analytical strategy

An inductive and semantic thematic analysis was conducted to construct global themes.^8^ Each transcript was read through multiple times and annotated by hand word-by-word to familiarize the researchers with the data in detail. Next involved ‘coding’ the raw data using keywords and phrases. Then similarities in perception, sex differences and patterns of talk were identified across codes, which led to initial themes. Initial themes were collapsed with other similar themes to arrive at global themes. This study analysis was triangulated among authors.

## Results

From the data, three main themes were constructed: (i) RISUG Hesitancy, (ii) Females perceived benefits of RISUG and (iii) Males perceived concerns regarding RISUG. The below themes present extracts as evidence to support the global themes.

### Theme 1 – RISUG hesitancy

While across the data set there was an overall positive response towards RISUG, participants did not feel everyone would be open to RISUG. Amy perceived the overall response would be a ‘*mixed bag*’. Jenna believed that people would be ‘*hesitant*’. Similarly, Joe claimed that there could be some ‘*hesitancy*’ and people, particularly males, would be ‘*cautious*’ towards something new due to vaccine hesitancy. This notion was also highlighted by Toby, that hesitancy towards RISUG could be a perception of newness and change, and Luke acknowledged that people would have to ‘*adjust*’ to it.

Emily argued that the different perceptions between the sexes could be the result of a division in ideologies:

And maybe it’s not based on whether they’re male or female [. . .] maybe [. . ..] both males and females could think, Oh, it’s the woman’s responsibility, because she’s the one that’s going to get pregnant. Or they might say, well, it’s the man’s responsibility as well. So, I guess it’s not necessarily going to be like, a gender divide, but maybe like a divide of like, ideology.

She suggests that males may think that it is a female’s responsibility to use contraception to prevent unplanned/unwanted pregnancy, while females think it’s males’ ‘responsibility as well’.

George raised perceived education levels would be a factor that determined hesitancy towards RISUG, associating hesitancy with lower educational attainment:

I feel like it depends on the person. I feel like a more educated person that understood the science and the whole process behind it [. . .] and if someone properly researched it, I feel like they’d be for it, compared to a person that doesn’t do the research will be against it because they’re not educated on the topic and people that aren’t willing to learn about it and aren’t willing to change, they’re the people that won’t take up on it.

What George further highlights is researchers and practitioners will have to learn how to relay education to the public more effectively on the contraceptive method to avoid hesitancy. One male participant related the hesitancy to vaccine hesitancy seen during the COVID-19 pandemic, as RISUG is also an injection.

### Theme 2 – Females perceived benefits of RISUG

Several of the females perceived that the one-off outpatient nature of the procedure was ideal. Jenna perceived RISUG would be more effective than long acting reversible female contraceptives:

You know, I have to take the pill or you know, my coil is running out or my implant is running out. So I have to get it changed. It’s just like a one-off procedure and it lasts a long, long time from what I read, like really good results.

Female contraceptive changes are a nuisance, she has ‘to get it changed’ by a health professional, where for males it would be injected and last a ‘long time’, with ‘good results’.

Katie also highlighted that another benefit of RISUG would be that there are few adverse side effects:

Just the lack of side effects. I think [. . .] the side effects of contraception is like such a big thing and can be so detrimental so the lack of side effects that have shown so far is definitely a benefit.

She perceives RISUG’s lack of side effects as novel and she suggests side effects to female contraception are ‘detrimental’.

### Theme 3 – Males perceived concerns regarding RISUG

Luke perceived other males might worry about the possible risk of long-term effects that may not have yet presented:

Because it’s so new, the long-term effects of using it is probably going to be my biggest hypothetical concern. It’s all well and good saying, oh, well, in these experiments, they showed zero side effects. But how long have subjects been exposed? Like, does it have an effect? 10 years down the line, does have an effect 20 years down the line? Obviously, we don’t have that research yet. And we won’t for a long time.

He perceives there will be difficulty in marketing without the long-term side effects being known, and that this will take years. However, his questioning can be used to inform effective health messages as RISUG has been in development for over 30 years.

Furthermore, Connor perceived the negative misconception that RISUG may create a way for people to ‘stealth’ individuals with greater ease. There is no way to check a man has indeed undergone the procedure, therefore he would ‘stealth’ or lie and say they have undergone RISUG to avoid wearing a condom during sexual activities:

I feel like some women would believe that and some men probably would say that they’ve had this injection when they haven’t just so they don’t have to wear a condom – . . . It opens up a whole new sort of element to a well you know, the term stealthing, and like a whole new side of that, really, because it’s the same thing essentially, isn’t it? It’s some sort of, or sex under false pretences or you know, deviate in a way from what was actually consensual.

He raises an important point that it is also important to consider how these procedures might be used for reproductive coercion and that this is perceived to be non-consensual behaviour.

## Discussion

Despite the positive perception from females and some males, most participants perceived there would be some form of hesitancy from the public if RISUG was to become available in the UK. Many of the participants argued that males would be more resistant than females. RISUG is a relatively new development, participants expressed the perceived novelty of RISUG would be the reason why some males may be hesitant to use the method. This is supported by Buck et al.,^5^ who also found males to be less willing to use a NH-IVIG as males might experience adverse effects.

Most of the male participants perceived several concerns regarding RISUG, such as the aforementioned long-term side effects, other concerns cantered around the inability to successfully reverse RISUG, the failure of successful administration and that it was an injection similar to a vaccine. Alternatively, there may be a general shift in male patient attitudes towards various hesitancy in seeking treatment for sensitive issues that involve genitals, as observed in cases of penile cancer during the COVID-19 pandemic.^9^ Despite the concerns, evidence suggests that RISUG is completely safe to use, there is little evidence to support the claim that reversal is possible, though trials in mice are underway.^4^ While administration error is a valid concern, in the most recent phase III trial, only 4.3% experienced administration error (through manufactory leakage or counter puncture error), lower than the failure rate of typical use condoms. Although the RISUG injections and the COVID-19 vaccine are completely different in their intended purposes, both nevertheless injections, resistance could then also partially be a result of any public concerns.

Another key concern was males engaging in non-consensual and coercive behaviours, such as ‘stealthing’.^10^ There is no research on the prevalence of ‘stealthing’ in the UK context to explain the likelihood of this behaviour. This warrants some consideration for potential risks to female partners. One possible solution is a medical certificate being provided should the patient who has undergone the procedure request it, with sensitivity placed to not roll out certificates as a mandatory part of care, as in some cultures this could be viewed negatively.^11^ Despite this concern, RISUG could be beneficial to help males gain some autonomy in family planning.

### Strengths and limitations

This study can begin to give insight into the perceptions of RISUG as a family planning method. The strength of this study is that interviews are a way to begin to gather in depth data to understand a social phenomenon. One limitation is that RISUG is currently unavailable in the UK and the themes are formed around prospective thoughts rather than actual perceptions of RISUG. Interviews with males in India who have been part of the trials could fill this gap. In addition, a larger sample of interviews can build off of the global themes from this exploration, using more advance qualitative methods such as Interpretative Phenomenological Analysis and Discourse Analysis to understand public perceptions. Another limitation is that the nature of the current study is qualitative, and it cannot be generalized to the wider society’s perceptions. For example, the researchers did not interview anyone who had vaccine hesitancy to understand their social acceptability of an injection based method of vasectomy. Further research on the topic is warranted before it becomes available on the market. Future research should also use quantitative methods to assess perceptions of RISUG, and human clinical trials should continue to assess the safety and efficacy of RISUG longitudinally.

## Conclusion

This exploratory thematic analysis suggests that further research is required to gain a larger corpus of data to understand public perceptions of RISUG. This exploratory thematic analysis suggests that trust of the method by the public can be valuable to improve the perceived acceptability of RISUG as a viable family planning method. A first step for researchers and practitioners would be to ensure effective education. The education should address the risks and benefits to improve both knowledge of the method and perceptions that is a viable and safe option, and delivered prior to the method reaching the contraceptive market.

## Supplemental Material

sj-docx-1-urj-10.1177_03915603241261813 – Supplemental material for A thematic analysis of the perceptions of reversible inhibition of sperm under guidance as a potential family planning method in the United Kingdom Supplemental material, sj-docx-1-urj-10.1177_03915603241261813 for A thematic analysis of the perceptions of reversible inhibition of sperm under guidance as a potential family planning method in the United Kingdom by Cristina M Gheorghe, Olivia Slack and Amanda D Wilson in Urologia Journal
