# Supplementary material for: A thematic analysis of the perceptions of reversible inhibition of sperm under guidance as a potential family planning method in the United Kingdom
Source: Urologia. 2024 Jul 26;91(4):819–23. doi: 10.1177/03915603241261813 (PMC11484156; doi:10.1177/03915603241261813)
Supplement: sj-docx-1-urj-10.1177_03915603241261813 – Supplemental material for A thematic analysis of the perceptions of reversible inhibition of sperm under guidance as a potential family planning method in the United Kingdom [file sj-docx-1-urj-10.1177_03915603241261813.docx]

**Supplemental Materials**

**Reversible Inhibition of Sperm Under Guidance (RISUG) INFORMATION SHEET**

Please read this information sheet prior to your interview. This will allow you to have a baseline understanding of RISUG prior to interviewing.

**WHAT IS RISUG?**

RISUG is an acronym and stands for Reversible Inhibition of Sperm Under Guidance and throughout this information, it will be referred to RISUG.

RISUG is a type of chemical contraception procedure for males which comes in the form of an injectable gel.

**WHAT IS RISUG MADE FROM?**

RISUG is formulated of two polymers (Styrene and Maleic Anhydride (SMA)), which is dissolved in a substance (Dimethyl Sulphoxide (DMSO)) which forms the injectable gel. This gel is extremely affordable to produce.

**HOW DOES RISUG WORK?**

This gel is injected into both of the tubes in which sperm passes through on their way from the testes to the penis.

Then, the gel forms a layer inside the walls of these tubes which causes partial blocking. This causes the gel to break down layers of mature sperm cells and act as an obstacle of the enzymes which are required to fertilize a mature egg from being released when sperm passes through the tubes.

So, this results in any ejaculation not containing any fertile sperm.

**WHAT IS THE PROCEDURE OF GETTING RISUG LIKE AND WHAT CAN BE EXPECTED?**

The procedure is a very quick (a few minutes) and non-invasive procedure which involves a local anaesthetic being applied to the scrotum and the receiving of one injection. Males will be able to resume normal to their normal sexual activity soon after.

RISUG claims to provide completely sterilisation from this single injection for up to 10-15 years, but can also be completely reversed, whenever desired, through the reception of a secondary reversal injection of water and baking soda which flushes out the RISUG.

Side effects appear minimal and may include swelling at the injection site.

**WHAT RESEARCH HAS FOUND**

There is only very early and unpublished research suggesting the ability of RISUG to be flush out, meaning that fertility is restored.

Some researchers observed changes of the shape/form of the tubes that RISUG is injected into within rats, but, but these changes return to normal once the RISUG was flushed from the tubes (Verma et al., 1981). Although these changes were not severe, and restored, it still highlights the newness and uncertainty that come with RISUG.

Research tested the toxicity of SMA on Rhesus monkeys and deemed a dose of up to 500mg of SMA polymer to be toxically safe in Rhesus monkeys, therefore it can be assumed from this that it should be safe for human use too (Sethi et al., 1990). The SMA component of RISUG is only 60mg dissolved within 120μL of DMSO (Lohiya, Alam, Hussain, Khan & Ansari, 2014), which is way below the 500mg maximum tested on the monkeys.

Human volunteers have taken part in Phase I, III and III clinical trials. The most recent Phase III clinical trials involved 139 male participants who already had a wife and at least two children. It was found from a six-month follow-up that all participants and their wives had normal health and experienced no severe negative side effects. Most individuals did experience scrotal swelling and pain, but this was temporary and disappeared within one month. 133 of the males experienced either an extremely low sperm counts or no sperm at all within their semen one month from the injection, with 82.7% of participants achieving azoospermia (semen containing no sperm) within two months, and the rest achieving this within three to six months. However, six participants did not achieve this due to administration error. Their findings indicate RISUG is an effective and safe method of family planning, but it also suggests administration must be done properly to ensure effective contraception. However, this study can be criticised due to limitations such as a very small sample size and a relatively short follow-up period duration. Moreover, this study did not attempt to reverse the effects of RISUG, so cannot provide any supporting evidence for RISUG’s ability to be reversed (Sharma et al., 2019).
